# Zigzag phosphorene nanoribbons: one-dimensional resonant channels in two-dimensional atomic crystals

**DOI:** 10.3762/bjnano.7.189

**Published:** 2016-12-13

**Authors:** Carlos J Páez, Dario A Bahamon, Ana L C Pereira, Peter A Schulz

**Affiliations:** 1 Faculdade de Ciências Aplicadas, Universidade Estadual de Campinas, 13484-350 Limeira, SP, Brazil; 2 MackGraphe -Graphene and Nano-Materials Research Center, Mackenzie Presbyterian University, Rua da Consolação 896, 01302-907, São Paulo, SP, Brazil

**Keywords:** 2D materials, constrictions, edge states, phosphorene nanoribbons, quantum dots

## Abstract

We theoretically investigate phosphorene zigzag nanoribbons as a platform for constriction engineering. In the presence of a constriction at one of the edges, quantum confinement of edge-protected states reveals conductance peaks, if the edge is uncoupled from the other edge. If the constriction is narrow enough to promote coupling between edges, it gives rise to Fano-like resonances as well as antiresonances in the transmission spectrum. These effects are shown to mimic an atomic chain like behavior in a two dimensional atomic crystal.

## Introduction

Low-dimensional systems have attracted attention over the past fifty years since the development of semiconductor epitaxial growth and deposition of metallic thin films [1]. The early scenario, back in the 1960s, as promising as it appeared, has evolved into a mainstream interest in condensed matter physics due to landmark discoveries in the late 1970s and early 1980s, such as the quantum Hall effect [2] and conductive polymers [3], respectively, 2D and 1D systems. The subsequent discovery of new carbon allotropes, showing stable structures either in 0D (fullerenes), 1D (carbon nanotubes) and 2D (graphene) consolidated this scenario in an exciting research field [4]. The isolation of strictly one atom thick layers in the first years of the present century opened a wider window for both basic physics and device applications [5]. These new disruptive research efforts, initially impulsed by graphene, are nowadays detaching from carbon-based roadmaps, as also envisaged, at the beginning of the graphene boost, by Novoselov, Geim and co-workers [6].

A very recent 2D atomic crystal of black phosphorous [7–10], namely phosphorene, is a promising system in which 2D properties together with strictly 1D chain behavior are present in different energy windows. This allows the same device to be tuned from a 1D to a 2D system by simply tuning the Fermi energy. In the present work we focus on the 1D energy window, created by an effective doubly degenerate band (in the relevant energy scale) associated to states strongly localized at the zigzag edges [11–12] of phosphorene nanoribbons, whose properties are explored using a new strictly one-dimensional resonant tunnelling device.

The double-barrier resonant tunnelling device [1,13–14], conceived here as an atomically precise segmentation at one of the edges, shows unusual geometry, since the direction of the barriers is perpendicular to the well and contact regions [15–16]. Among our findings we show that for a thin barrier case (constriction with narrow step from the upper zigzag edge), the resonant tunnelling permits a spectroscopy of the band structure of phosphorene nanoribbons in this energy window. Furthermore, progressive widening of the barriers (enhancing the step width of the constriction), thus nearing the constriction to the other edge leads to edge-coupling effects featuring resonances with Fano line shapes [17–19]. Also, a new discrete/continuum-states coupling system is revealed. For this latter coupled-edge system, the transmission probability characteristics turn out to present clear features of both (i) the actual finite confining segment coupled to an infinite (not segmented) edge and, (ii) the properties of an infinite narrow nanoribbon with strongly coupled edges. These results are independent of the area of the device region. They solely depend on the length of the segmented region and distance between the edges, revealing an effective chain-like behavior of the edges of the nanoribbons.

In what follows, we initially discuss the “bulk” electronic properties of a phosphorene nanoribbon. We present the model calculation framework, as well as the effects of edge coupling on the conductance of these infinite zigzag ribbons, which are essential to understand the resonant tunnelling spectra. Next, the geometry of the actual investigated segmented device is presented, introducing the resonant tunnelling effects. Subsequently, the core of the results is devoted to explore the resonant tunnelling behavior of the segmented-edge device, showing how the defects on the edge may actually enrich the scenario instead of solely washing out the announced effects. This provides further evidence that the phenomenon is restricted to the atoms at the very edge. Finally, the conclusion suggest a bridge between the present 1D systems embedded in a 2D crystal and ongoing research on isolated atomic chains.

## Results and Discussion

### Phosphorene zigzag nanoribbons and model calculation: edge-coupling effects

**Figure 1:**
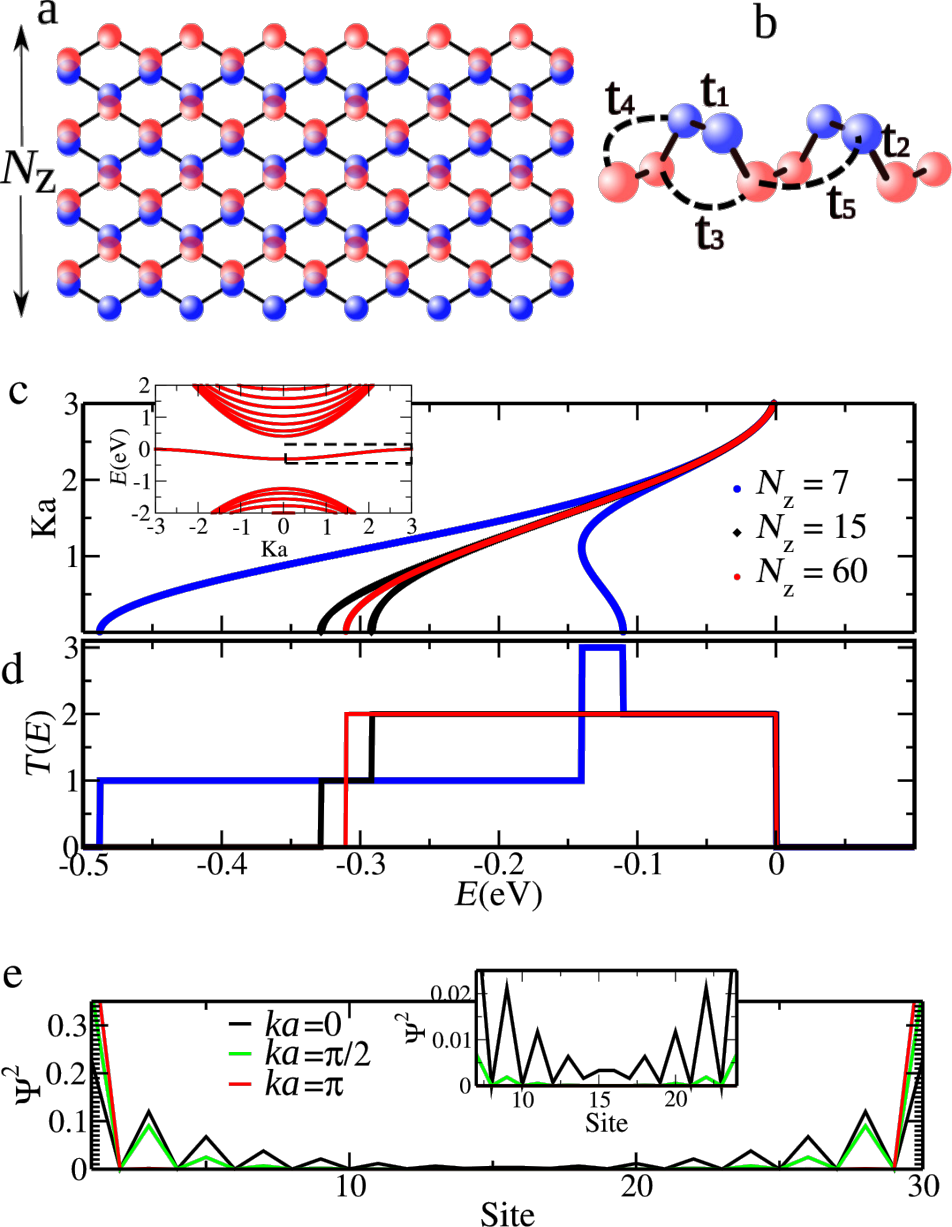
(a) Illustration of a zigzag phosphorene nanoribbon of width *N_Z_*, where *N_Z_* is the number zigzag chains. (b) Hopping parameters used in the four-band model [20]. (c) Structure of the central band of edge states of nanoribbons with different widths. The inset includes the band structure of the bottom and top of the conduction and valence bands for *N_Z_* = 60. (d) Transmission probabilities for the edge states depicted in panel (c). (e) Probability amplitude of edge states for *N_Z_* = 15 for different values of *ka*. The inset reveals the stronger non-zero overlapping of states of the two edges at the bottom of the central band, *ka* = 0.

The essential atomistic aspects of the structures investigated are depicted in Figure 1. Figure 1a shows a segment of an infinite zigzag edged phosphorene nanoribbon of width *N_Z_* = 8, which is the number of zigzag chains along the ribbon. The tight-binding hopping parameters considered, as discussed below, are indicated in Figure 1b.

The quite complex electronic structure of phosphorene, already at energy ranges rather close to the Fermi energy, hinders a wider use of single-orbital tight-binding models in chasing the alluded electronic and transport properties of systems based on this new material. Nevertheless, the use of such model is well validated, by means of comparisons with first-principle electronic structure calculations [20–21], for the very energy window of interest around the gap, namely a double central band. This central band for zigzag phosphorene nanoribbons has been predicted for phosphorene [11–12] and is absent in graphene. We use here the same tight-binding parametrization for phosphorene proposed by Rudenko [20] considering a Hamiltonian 
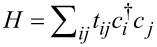
, where *c_i_* (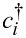) is the creation (annihilation) electronic operator at site *i* and *t_ij_* is the hopping integral between sites *i* and *j*. In this model, five hopping integrals are required to characterize the low-energy electronic properties [20]: *t*_1_ = −1.220 eV, *t*_2_ = 3.665 eV, *t*_3_ = −0.205 eV, *t*_4_ = −0.105 eV and *t*_5_ = −0.055 eV. The transmission *T* = Tr[Γ*_L_G^r^*Γ*_R_G^a^*] is calculated using the recursive Green’s function [22] *G^r^* = [*E* + *i*η − *H* − Σ*_L_* − Σ*_R_*]^−1^ in the phosphorene lattice representation. The broadening function of the left and the right contact 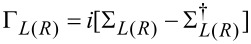 and the self-energy of contact Σ*_L(R)_* are calculated recursively for the semi-infinite zigzag phosphorene nanoribbons [23]. Other electronic properties such as the local density of states (LDOS) 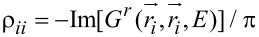 are also calculated.

The electronic and transport properties of a host zizgzag nanoribbon, in which a finite segment will be latter tailored in, are also summarized in Figure 1. The inset in Figure 1c depicts the energy window of interest, showing the top (bottom) of the valence (conduction) band and an effectively degenerate central band [11]. These central bands present cosine-like dispersions characteristic for 1D systems [11]. Indeed, the degeneracy comes from the fact that the width of the ribbon here is *N_Z_* = 60, which guarantees that the two edges are effectively uncoupled [24]. Hence, this width will be chosen for the host ribbon where the constriction will be introduced.

The effect of edges coupling with the band structure can be followed in the main part of Figure 1c, showing a zoom of the central band energy range. Having in mind the uncoupled limit of *N_Z_* = 60 (red curve), lifting of the central band effective degeneracy starts (in the present relevant energy scale) for *N_Z_* = 15 (black curve) at the center of the Brillouin zone with an approximately symmetric splitting of slightly deformed cosine-like bands. Indeed an incipient overlap of wave functions in this situation is illustrated in Figure 1e, with a noticeable amplitude of the wave function in the atomic sites well inside the ribbon. It should be note that *N_Z_* corresponds to the number of zigzag chains, hence for *N_Z_* = 15, there will be 30 atomic sites in the unit cell. For extremely thin ribbons, *N_Z_* = 7, the splitting attains values of the order of the uncoupled band widths (blue curve). More striking is the drastic change in the shape of one of the bands, showing a local maximum at the center at *ka* = 0 and a minimum at *ka* = 1.

The consequences of the edge coupling on the transmission probabilities along the edges are qualitatively significant as can be seen in Figure 1d. Degenerate bands sum up to a plateau of *T* = 2 (red curve). A slight lifting of the degeneracy breaks the lower threshold of the plateau introducing a *T* = 1 step, an energy range where there is only one conduction channel [11]. However, extreme coupling leads to a *T* = 3 plateau for −1 *< ka <* 1, where the different states corresponding to the same energy in this band are added to the channel associated to the other cosine-like band.

### Segmented nanoribbons: resonant tunnelling in 1D effective-chain structures

**Figure 2:**
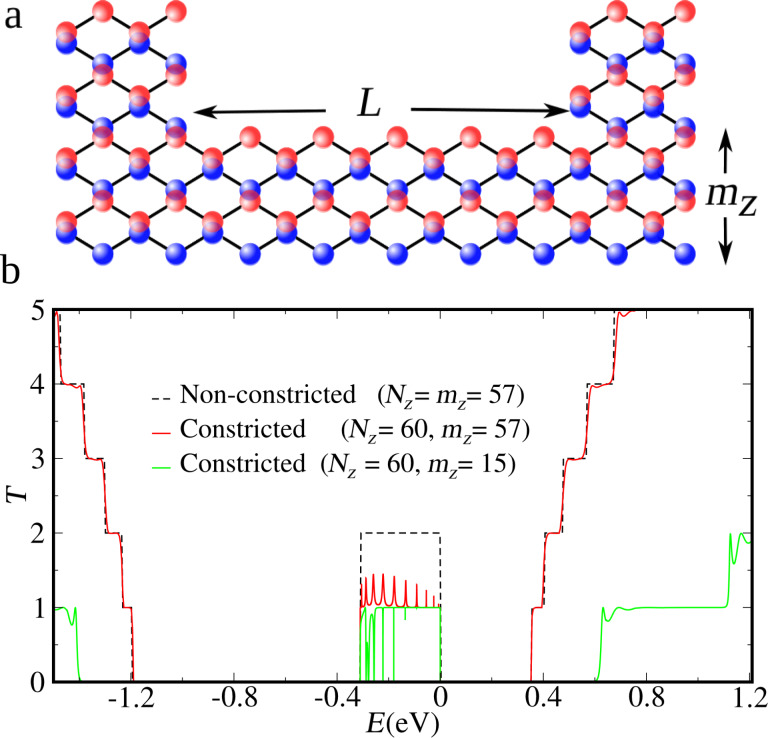
(a) Schematic picture of a constriction characterized by the parameters *L* = 10, actually used throughout the work, and *m_z_* = 4, here only for the sake of illustration. (b) Transmission probabilities, as a function of the Fermi energy, at the energy range corresponding to the central band, including the bottom and top of the conduction and valence bands. Three different situations are depicted: two constriction defined at nanoribbons of width *N_z_* = 60, both with *L* = 10, and *m_z_* = 57 (red) and *m_z_* = 15 (green) and a zigzag nanoribbon without any constriction, *N_z_* = *m_z_* = 57 (black line).

In the energy energy range of the edge-states band, the “bulk” of the nanoribbon acts mainly as “in plane” substrate for the two-dimensional channels at the edges. This condition, evidenced by the electronic band structure discussed in the previous section raises the question of a means to observe experimentally those effective one-dimensional chains embedded in the rather complex phosphorene crystalline structure. In order to test our hypothesis we propose the segmented nanoribbon structure (constriction) illustrated in Figure 2a. The segment of a thinner region of the nanoribbon of width *m_Z_*, the number of zigzag chains, is defined by a length *L* in units of atoms removed along one zigzag direction. One essential parameter is the step width between the semi-infinite upper edges and the central segment, which is simply defined as *N_Z_* − *m_Z_* and, as will be seen below, defines the barrier thickness in the resonant tunnelling.

Figure 2b shows the transmission probabilities through two constrictions of length *L* = 10 with *N_Z_* − *m_Z_* = 3 and *N_Z_* − *m_Z_* = 45 step widths, compared to a bare *N_z_* = 57 nanoribbon, as a function of the energy. In order to avoid any coupling between the edge states the width of the nanoribbon in the contacts is also *N_Z_* = 60. Transmission plateaus above and below the edge states band are shown, for the sake of completeness, since these structures are of entirely different character than the resonances in the central band we will be focusing on. These transmission plateaus are due to the lateral confinement in a nanoribbon. This is confirmed by the green curve for *m_Z_* = 15, a deep step leading to a large shifting of the valence and conduction bands transmission plateaus, evidencing also the well-known Fabry–Perot oscillations [25] due to the geometrical modulation. These effects are already well known for graphene and square lattice nanoribbons with constrictions [25–29].

**Figure 3:**
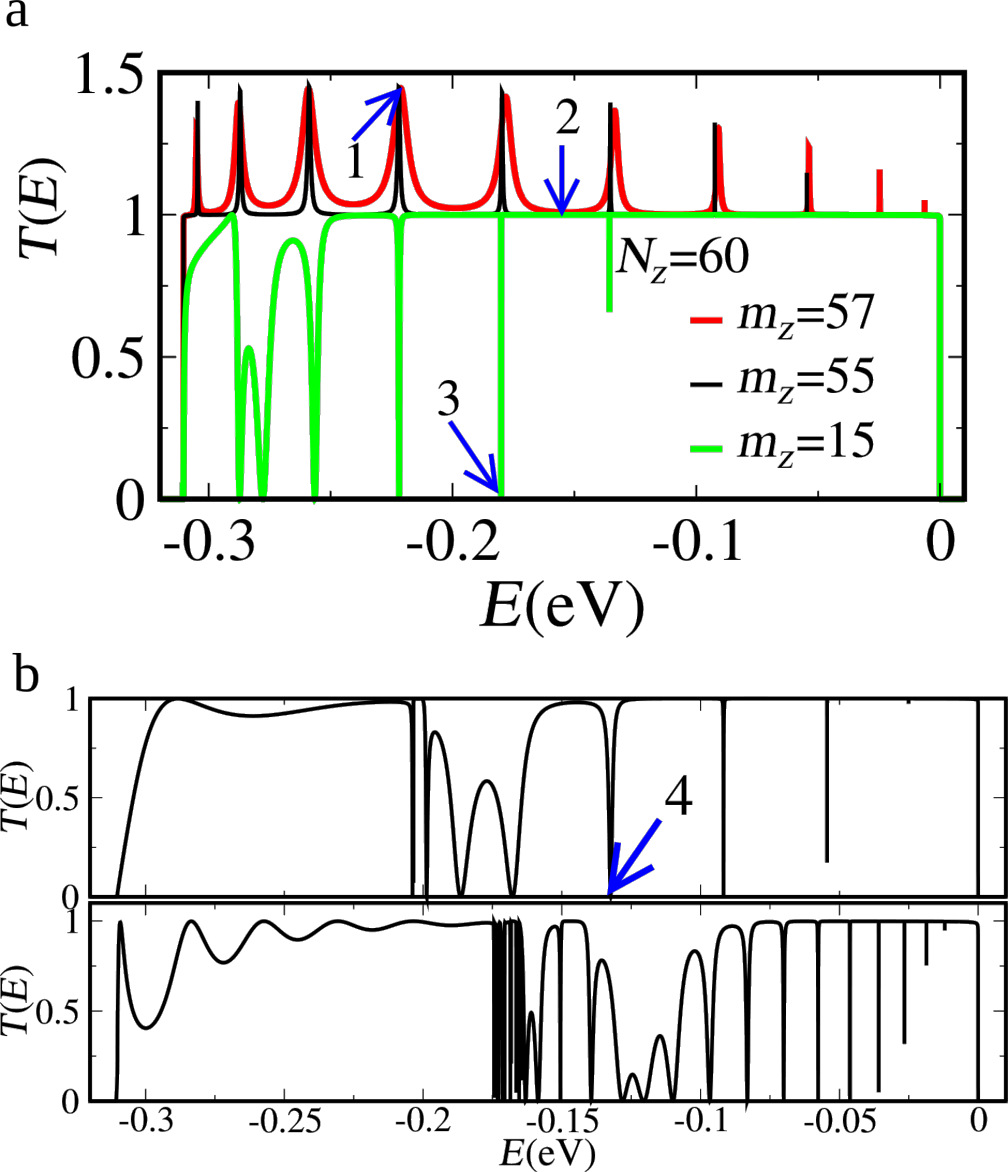
(a) Transmission probabilities, as a function of the Fermi energy, in the energy range of the central band corresponding to nanoribbons with constrictions. Red and green correspond to the cases in Figure 2b, *N_Z_* = 60, all with *L* = 10 and *m_Z_* = 57 (shallow constriction) and *m_Z_* = 15 (deep constriction), respectively. The black curve corresponds to a constriction with *m_Z_* = 55 (intermediate depth). (b) Transmission probabilities for very deep constrictions, *m_Z_* = 8 and different lengths: *L* = 10 (upper panel) and *L* = 30 (lower panel).

The edge states, observed here in the energy range from 0.3 to 0 eV, drop to *T*(*E*) =1 with resonant peaks on top for wide constrictions and anti-resonances for narrow constrictions. Figure 3a presents a closer look at this energy range. For thin armchair steps (*N_Z_* − *m_Z_* = 3) the red curve shows a group of 10 peaks, these resonances get thinner as the step increases (*N_Z_* − *m_Z_* = 5).

When *m_Z_* is further diminished, the barriers to the upper-edge contacts become too large, but now the coupling to the lower edge becomes relevant. For *m_Z_* ≤ 15 transmission shows asymmetric Fano-like resonances at the low-energy side and sharp anti-resonances at higher energies within the central band, which will be discussed below. It should be recalled here that only for extremely thin nanoribbons the strong coupling between the edges widens the central band. However, a plateau enlargement for *m_Z_* = 8 is not observed in Figure 3b, because the edges of the left and right contact are not coupled (keeping the central band of the contacts unaltered).

**Figure 4:**
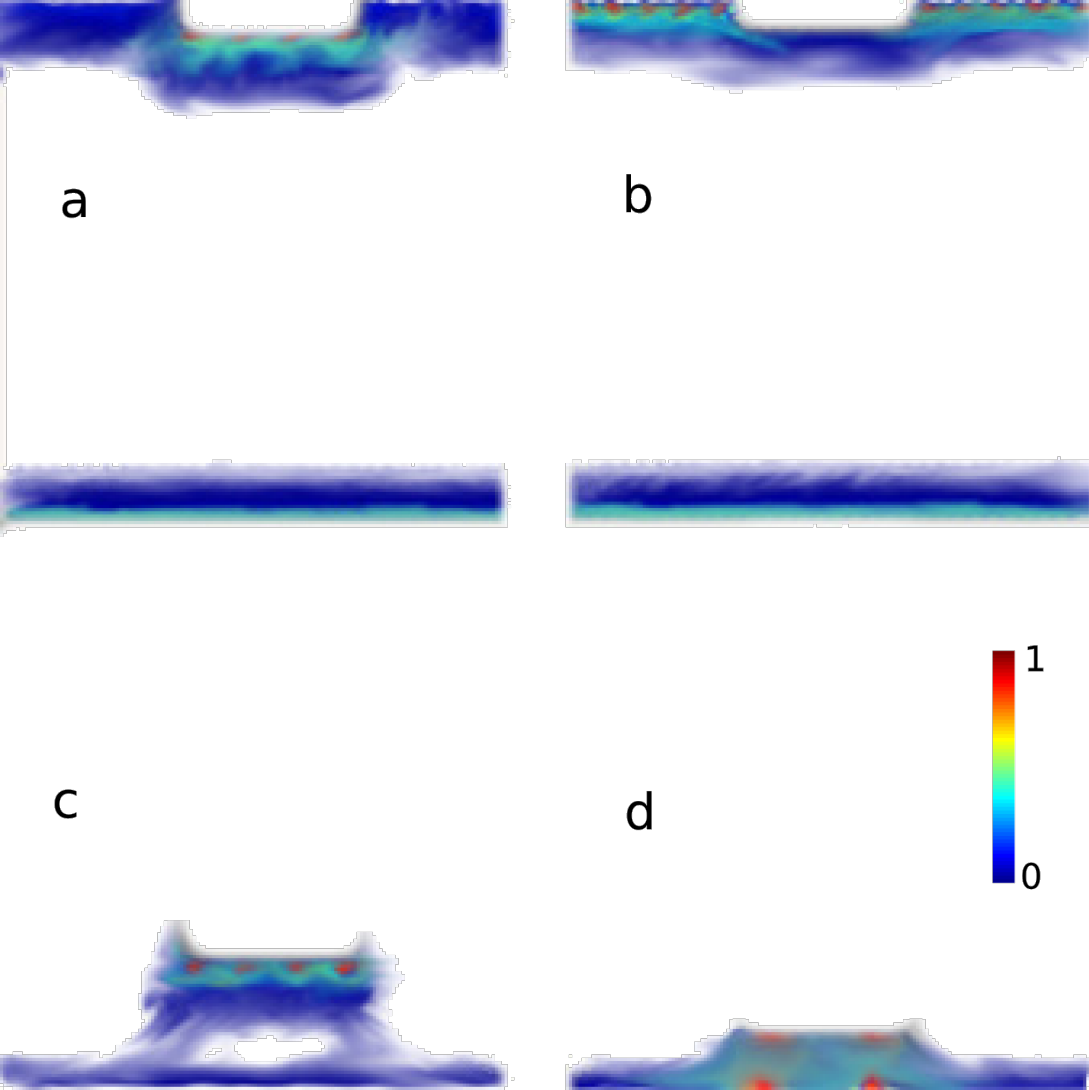
LDOS at the energies pointed out by the arrows, labeled 1, 2, 3 and 4, in Figure 3 for *L* = 10. The upper panel is for shallow constrictions, *N_Z_* = 60 and *m_Z_* = 57: (a) at resonant energy, corresponding to arrow (b); at off-resonant energy, corresponding to arrow 2. The lower panels are for deep constrictions: (c) at the anti-resonance indicated by arrow 3 (*m_Z_* = 15); (d) at the anti-resonance highlighted by arrow 4 (*m_Z_* = 8).

In our constriction the role of both channels (discrete states and continuum at the upper and lower edges) can be made explicit by picturing the local density of states (LDOS) in Figure 4, at the energy values indicated by arrows 1, 2, 3 and 4 in Figure 3. In Figure 4a, looking at the LDOS corresponding to a transmission peak pointed out by the arrow 1, it is clear that the higher values of LDOS appear on the edges. The confined state at the constriction in the upper edge clearly stands out. It should be noted that this LDOS is slightly asymmetric, since the structure with *L* equal to an even number of atoms is asymmetric (see Figure 2a). This asymmetry leads to a resonance peak *T <* 1 [30] superposed on the background plateau. *L* equal to an odd number of atoms restores complete symmetry, leading to higher resonances, *T* ≈ 1 (not shown here). A less intense LDOS along the lower edge corresponding to the *T* = 1 plateau can also be observed. The LDOS along the upper edge outside the constriction region is less intense in the figure scale, due to the prominence of the confined state. Far from a resonance, actually between two resonances, is the situation pointed out by arrow 2 in Figure 3a. The LDOS in the confined part of the upper edge is strongly suppressed, enhancing the contribution along the entire lower edge and the upper-edge contact sections (i.e., outside the confining region, Figure 4b). A quite different situation is depicted in Figure 4c,d, where the confined state in the constriction is decoupled from the upper edge with a variable coupling to the lower edge. In Figure 4c, corresponding to the anti-resonance labeled 3 in Figure 3, we observe a faint coupling to the lower edge.

**Figure 5:**
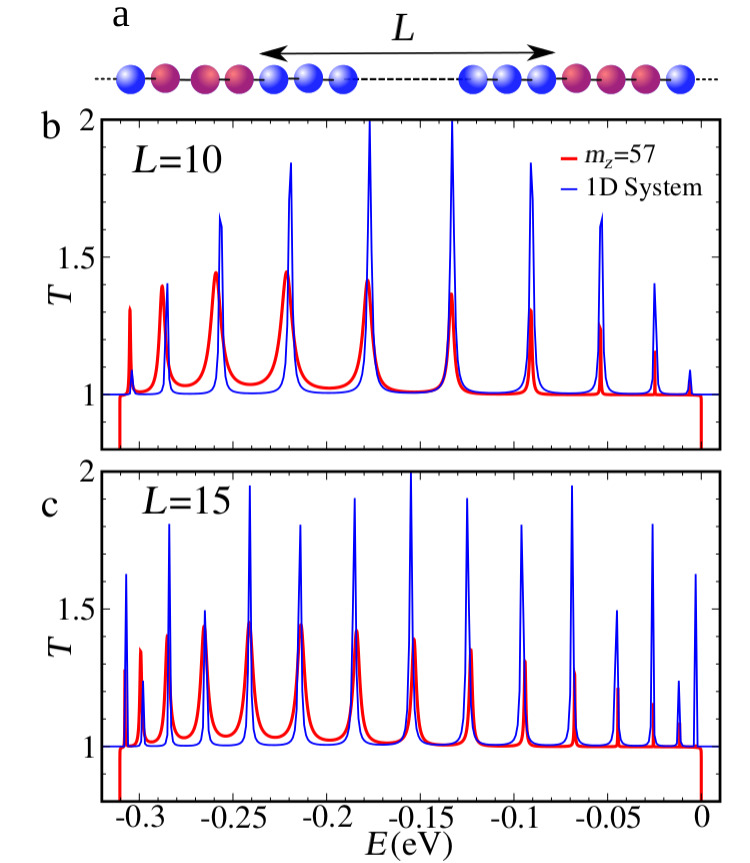
Comparison between the transmission probabilities at the central band-energy ranges for shallow constrictions, *m_Z_* = 57, and equivalent s-like orbitals chain toy model. (a) Representation of the one 1D double-barrier quantum well structure at the upper edge. The central blue sites represent the quantum well at the constriction, while the red ones are for the barriers (armchair steps). The left and right contacts at the upper edges are also represented by blue sites. (b) Transmission probabilities for the constriction *L* = 10: 4-band tight binding model (red) and the toy model (blue). (c) The same as (b) but for a longer constriction, *L* = 15.

The LDOS plots reveal the unique character of the resonances observed in Figure 3, namely resonant tunnelling through confined edge states in the constriction defined by an armchair-like step double barrier structure. It should be noted that the number of resonances is identical to the number of atoms removed along the segment that define the length of the constriction, *L* = 10. This indicates that the transmission shows a spectroscopy of the 1D states at the edge of the constriction. Indeed, increasing the length of the constriction will increase at the same proportion the number of the resonances (not shown here). The fact that the resonances are insensible to the constriction width (hence the area for a fixed length) is a further indication that we are dealing with a strictly 1D effect at the edges. Albeit there is the underlying 2D crystal, the behavior revealed here is the one of an effective atomic chain. Here we should note that the only signature of the underlying 2D crystal is given by the resonances widths. Recalling Figure 1e, the resonances near the bottom of the central band, *ka* ≈ 0, correspond to states that penetrate deeper into the bulk, hence the barriers defined by the device steps are less effective than for resonances at higher energies. Two-dimensionally structured systems, such as nanoribbons, present transmission probabilities with multi-channel contributions that are mixed by the geometrical changes along the structure [31–32]. On the other hand, one-dimensional systems present single-channel transmission probabilities, that are described by s-like orbital chain models. The positions of the transmission resonances are shown in Figure 5 to be well reproduced by a simple one-dimensional double-barrier quantum well modeled by a chain of s-like orbitals [32–33]. The nearest neighbor hopping (*t*_1D_) of the one-dimensional chain, shown in Figure 5a, is calculated by |*t*_1D_| = Δ*E*/4 = 0.0775 eV, where Δ*E* is the edge-states band width obtained from the red curve in Figure 1c. The transmission peaks of the one-dimensional quantum well with *L* = 10 and *L* = 15 atomic sites clearly reproduce the position of the resonances observed for phosphorene constrictions of the same length (Figure 5b,c). The resonant peaks appear at the energies of the infinite square-well energies *E_n_* = 2*t*_1D_ + 2*t*_1D_ cos(*n*π/(*L* + 1)) for *n* = 1, 2,…, *L*.

**Figure 6:**
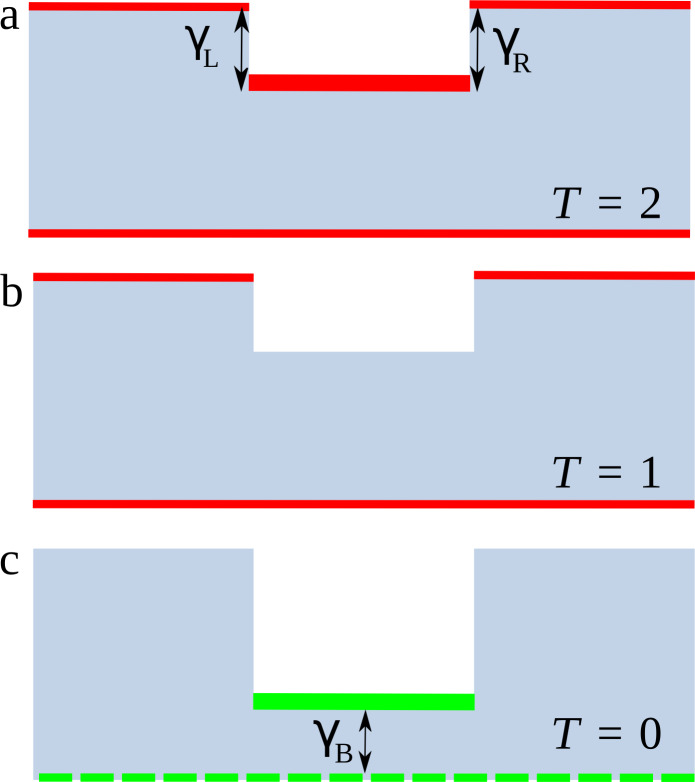
Schematic representation of the main transmission-probability conditions. The grey areas represent the nanoribbons with constrictions, with different edge-state behaviors highlighted by red and green lines. (a) Resonant transmission at the upper edge summed up to the continuous transmission at the lower edge, hence *T* = 2. The resonant coupling of the confined state at the constriction with the left(right) contact is represented by γ*_L_* (γ*_R_*). (b) Off-resonance negligible transmission at the upper edge with the continuous transmission at the lower edge, *T* = 1. (c) Anti-resonance in the transmission due to the strong coupling, represented by Γ*_B_*, between the confined state at the deep constriction and the lower edge, now depicted as a dashed line indicating the absence of transmission, *T* = 0.

To recap, the edge states at opposite edges provide one-dimensional electronic transport channels embedded in a two-dimensional material and the conductance observed can be understood from a simple model. The resonant peaks on top of the *T* = 1 plateau resemble the conductance of two parallel and independent channels, as shown in Figure 6a. The lower edge provides a continuous channel of *T* = 1 while the upper edge presents a quantum well with tunnelling coefficients γ*_L(R)_* across the left (right) barrier defined by the vertical edges in the figure. Hence, resonant tunnelling becomes only possible when the energy matches the energy of the bound states in the well. When this situation is not fulfilled the upper channel is closed and the transmission of the whole system is *T* = 1, see Figure 6b. A deeper step on the constriction, on the other hand, would lead to quasi-bound states, i.e., wider barriers, connected to the upper edge contacts, but with a significant coupling, γ*_B_*, to the bottom edge, Figure 6(c), leading to Fano-like resonances and anti-resonances in the transmission [34–35]. This situation corresponds to quasi-bound states coupled to a continuum, leading to Fano-like asymmetric resonances and anti-resonances in the transmission.

The three different line shapes can be described by a single expression [18]:

[1]
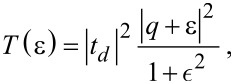


where *t_d_* is the direct transmission without the presence of a scattering region, ε = (*E* − *E_R_*)/Γ, (*E_R_* is the energy of the resonant discrete state and Γ the corresponding line width) and *q* is the Fano asymmetry factor, which represents the ratio of the resonant tunnelling channel to the channel due to the continuum (here represented by the lower-edge channel). When *q*→∞ and *t_d_*→0 (no continuum channel available) a resonance peak develops. For *q* ≈ ±1 (both channels are relevant), an asymmetric line shape is revealed, while for *q* = 0 an anti-resonance appears.

Recalling the framework of the present work, edge-confined states are supported only by zigzag edges and are absent in armchair or bearded edges [11–12]. Therefore, introducing perturbations to a zigzag edge, such as edge vacancies, would locally destroy these 1D states. The consequences of these perturbations are very relevant in the present early stage of the experimental development of phosphorene, in which only preliminary steps toward design and realization of effective devices from the bulk onto the nanoscale have been reported so far [36]. Hence, effects of disorder at the edges have to be considered.

**Figure 7:**
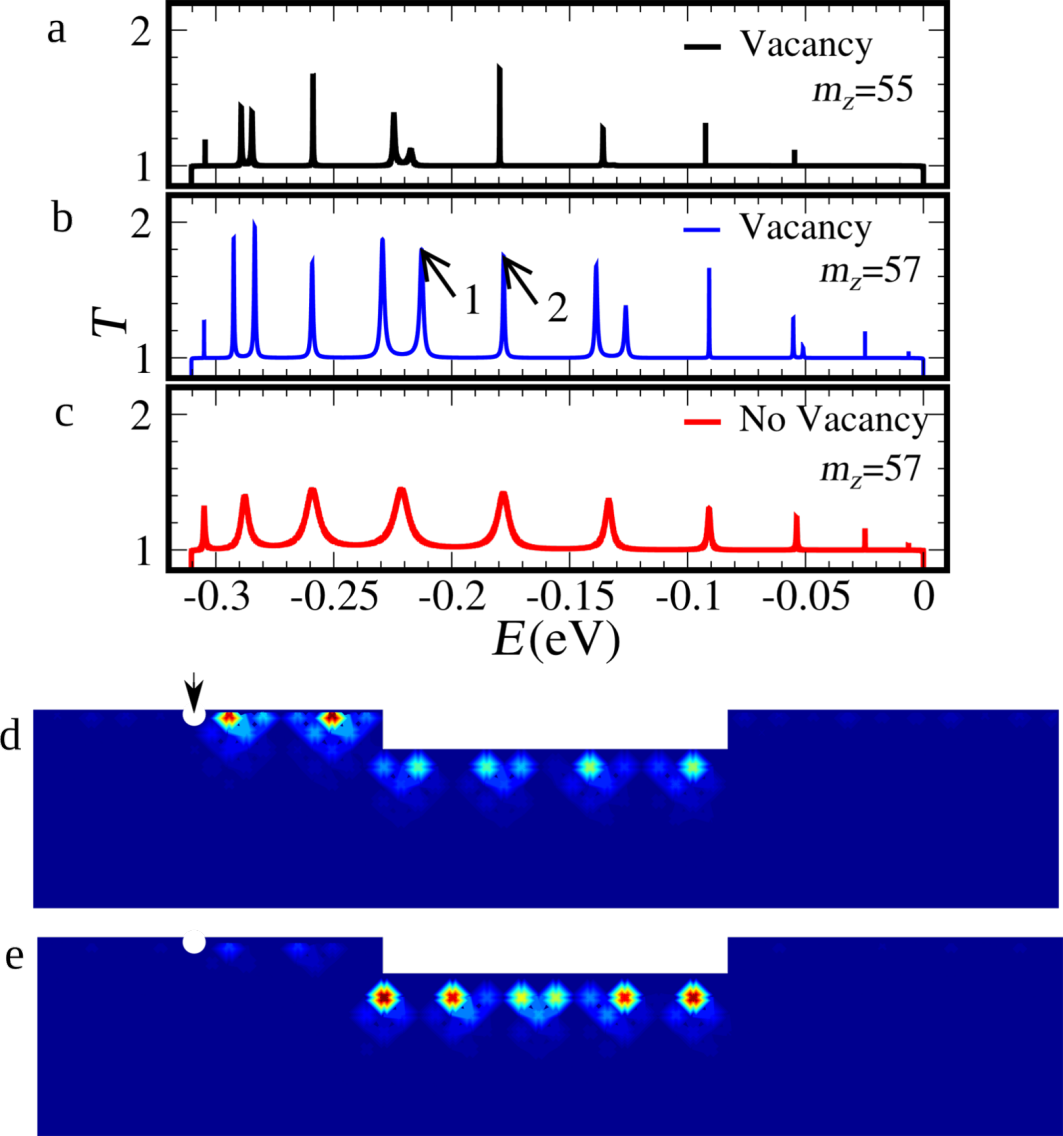
Edge states transmission probabilities for a nanoribbon *N_Z_* = 60 wide, with a constriction *L* = 10 long, adding an edge vacancy at the left contact: (a) *m_Z_* = 55 and (b) *m_Z_* = 57. (c) The system without vacancies for the sake of comparison, highlighting the splitting of the resonances due to the presence of a vacancy. (d) LDOS at the energy indicated by arrow 1 in panel (b). (e) LDOS at the energy indicated by the arrow 2 in panel (b).

In Figure 7 we present the transmission probability as a function of the energy as well as the LDOS associated to selected resonances in the presence of vacancies. Defects are normally seen as mechanisms that hinder the observation of transport properties associated to shape modulation of nanoscopic low-dimensional systems. Indeed, the resonance spectra are also dramatically modified in the present case. However, the issue can be seen from an entirely different point of view. The vacancies change locally the character of the edge. Thus, they actually introduce small barriers and further divide the system into smaller segments. The system chosen here is a device with a vacancy located at the upper edge of the left contact near the central segment (quantum well), the exact position is marked by the arrow in Figure 7d.

What can be observed from the transmission probabilities in Figure 7a–c is that the resonances of the well at the contact defined to the left by a barrier due to the vacancy, couple to some of the states of the original well given by the central segment. Those couplings are identified by the split peaks clearly seen in Figure 7b. If the barriers defining the central segment (well) are widened, Figure 7a, the splitting diminishes. Figure 7c depicts the same device without the vacancy as a guide for identifying the couplings. The character of the one-dimensionally confined states in the presence of a vacancy is illustrated in the LDOS in Figure 7d and Figure 7e for the resonances corresponding to arrows 1 and 2 in Figure 7b, respectively. State 1 corresponds to a double-well (vacancy-upper edge contact-left step-central segment-right step) along the upper edge, exhibiting LDOS at both wells, while state 2 is confined mainly to the central quantum well.

Taking into account the previous discussion, resonant tunnelling spectroscopy would become rather involved with the presence of defect-induced barriers. However, scanning probe microscopy remains a way to reveal the edge quantum confinement.

## Conclusion

So far, we have ignored the effect of Coulomb interactions. It is well known that the interplay of Coulomb blockade and quantum confinement in quantum dots leads to rich physical phenomena [37]. The correlation among the energy scales involved such as energy-level spacing of the constriction, charging energy and couplings allows us to estimate when charging effects would be important [38]. In the strong-coupling regime the wave functions of electrons in the shallow/deep constriction and the wave function of electrons in the upper/lower edge greatly overlap washing out the Coulomb blockade. Tunnelling cannot be sequential and transmission is dominated by eigenstates of the constriction [39]. On the other hand, when the constriction is weakly coupled to the upper and lower edges (weak-coupling regime) charging effects can be significant depending on the length of the constriction. In addition, metallic leads, gate electrodes and the dielectric function of the substrate modify the capacitance of the constriction and therefore a detailed study of the charging effects is left for future work.

It is inevitable to compare our results with those obtained for graphene constrictions. Both zigzag graphene and phosphorene nanoribbons support edge states. However, their signatures on the electronic transport properties are completely different. First, edge states in zigzag graphene nanoribbons are sublattice polarized, so one single edge do not contribute to the electron transport properties. The graphene edge states channel originates from the overlapping of edge states on opposite edges [40], contrary to what we observe here where a single phosphorene edge provides an independent transport channel. Second, localized states in graphene junctions manifest as anti-resonances of zero conductance, these states are localized over the junction [26–28]. The localized states of phosphorene are constricted to the grooved zigzag edge and appear as peaks, asymmetric Fano line shapes or dips in the conductance. In summary, we propose phosphorene zigzag nanoribbons as a platform for constriction (segment) engineering. In the presence of an engraved segment at the upper edge, quantum confinement of edge-protected states reveals resonant tunnelling transmission peaks if the upper edge of the host nanoribbon is uncoupled to the lower edge. Coupling between edges in thin constrictions gives rise to Fano-like resonances and anti-resonances in the transmission spectrum of the system. One could envisage to observe these effects by means of transport measurements as well as scanning probe microscopy [41]. The energy scale given by the resonance spacing is of the order of 10 meV for constriction lengths of *L* = 30 (not shown here), corresponding to about 5.05 nm and contacts about 13.3 nm wide. This figures can count as a benchmark for experimental efforts, recalling that defects may lead to more complex spectra without washing out the main features. Other resonant tunnelling mechanisms have been observed in phosphorene nanoribbons with vacancies [42], defects [43] and transverse electric fields [44]. It is important to reinforce that these mechanisms involve states at the interior of the nanoribbon, whereas the effect shown here requires one-dimensional states localized at the edges.

Concomitant to the development of the fascinating physics of 2D materials, new extreme 1D systems, namely isolated atomic chains, either based on carbon [45] or metallic elements [46], have been obtained and characterized, with their properties and possible applications theoretically investigated. The present results suggest a way to obtain effective atomic chains from the edges of a 2D crystal.
